# Herman Berendsen, an Unforgettable Man

**DOI:** 10.1007/s10930-023-10099-4

**Published:** 2023-03-08

**Authors:** Anton J. M. Schoot Uiterkamp

**Affiliations:** Environmental Sciences, Integrated Research On Energy, Environment and Society (IREES), Energy and Sustainability Research Institute Groningen, University of Groningen, Groningen, The Netherlands

**Keywords:** Water, NMR, EPR, Copper ions

## Abstract

My memories of professor Herman Berendsen cover roughly two periods during which I had many contacts with him. Between 1966 and 1973 I was his MSc student and later his PhD student in the Department of Biophysical Chemistry at the University of Groningen. The second period started in 1991 when I returned to the University of Groningen as a professor of environmental sciences.

My memories of professor Herman Berendsen cover roughly two periods during which I had many contacts with him. Between 1966 and 1973 I was his MSc student and later his PhD student in the Department of Biophysical Chemistry at the University of Groningen. The second period started in 1991 when I returned to the University of Groningen as a professor of environmental sciences.

## Herman the Man

Herman was generous and resourceful. The day in June 1969, on which I was to receive my MSc degree in the Academy Building in Groningen, happened to fall in a week Herman and I participated in a biophysics summer school at the University of Twente in Enschede about two hours by car from Groningen. Since both of us wanted to attend the 11.00 h lecture by the famous Harvard biophysicist Elkan Blout Herman decided we should fly to Groningen. An exciting idea since I had never flown before. Moreover he offered to pay for my trip. After the one hour lasting lecture we raced to Twente Airport and flew in 20 min to Groningen-Eelde Airport in a twin engine turboprop Fokker F 27. At 14.00 h sharp Herman and I were sitting in the Faculty room in the Groningen University Academy Building opposite each other and surrounded by my fiancée, family members and friends. At 15.30 h Herman flew back to Twente Airport. Needless to say he missed my graduation party.

Herman enjoyed playing with language. At the end of his graduation eulogy he invited me to sign my MSc certificate saying “Wilt u dit *links onder*tekenen teneinde het *rechts*geldigheid te verlenen ?” (a Dutch language pun which is difficult to translate literally but sounds a bit like “please sign this in the lower *left* corner in order to make it *rightly* valid)*.* He also liked quoting palindromic sentences like “Madam I’m Adam”.

Herman was by far the brightest and smartest person I encountered during my professional career. At the same time he was also modest and unassuming and he gave his MSc and PhD students much freedom to pursue their own research interests. I wanted to study the copper binding site in the oxygen carrying protein hemocyanin from the snail *Helix Pomatia*. The structural characteristics of this very large multi-subunit protein had been actively studied for several years in the biochemistry department, but insight in the active site was still lacking. Although Herman’s main interests were in investigating water in biological systems he allowed me to start an EPR study of copper ions in hemocyanin.

Moreover he took care of providing me with a research position at ZWO (Netherlands Organization for Pure Scientific Reseach).

Characteristically he did not want to be a co-author of my first paper [1]. He argued that the key finding of the paper, the first observation of magnetic dipolar interaction between two copper ions in a protein, was mine exclusively and that I alone should take the credit for it. By doing so he taught me an important lesson that I applied myself later on when I had to decide on whether or not I should be a co-author on a publication of one of my students or coworkers. As a result Herman and I have only one publication together [2].

I finished my PhD thesis in the year 1972, the year in which the ground-breaking book “The Limits to Growth” was published and the first UN Conference on the Human Environment was held in Stockholm. Whether Herman foresaw that I would later on become an environmental scientist I don’t know, but after defending my PhD thesis in March 1973 he presented me with a copy of the iconic “Whole Earth Catalogue”.

I left Groningen in April 1973 and for almost twenty years Herman and I did not meet each other. In October I991 I returned to the University of Groningen as a professor of environmental sciences. Consequently from 1991 till 2019 I was Herman’s colleague as a professor and later as an emeritus professor at the Faculty of Mathematics and Natural Sciences (later renamed Faculty Science and Engineering) of the University of Groningen.

Since we were working in different departments and in different buildings we did not meet very often except at the Physics Colloquia where we were both regular visitors. Herman often expressed his interest in climate change and renewable energy, some of the topics my department was working on. Herman retired in 1999.

I distinctly remember two events from the period 1999 till Herman’s death in 2019.

Herman knew that I was a member of the Governmental Committee on Biobased Economy. He asked me if I could give his grandson Tim and his class mate some advice on the prospects of bioengineering for their high school graduation thesis. I spoke for over an hour with the two very tall young men (even taller than Herman) while a very proud and admiring grandfather Herman was watching us from the end of the table. It was Herman the family man I had never met before.

In 2013 the Nobel Prize in Chemistry was awarded to Martin Karplus, Michael Levitt and Arieh Warshel for “the development of of multiscale models for complex chemical systems". Together with many colleagues I felt that Herman should have been a recipient of the Prize as well given his fundamental contributions to the field of Molecular Dynamics (MD). Nevertheless the characteristically generous and modest Herman gave an outstanding overview of the contributions of the three Prize winners at the 2013 Nobel Prize Symposium organized by the Royal Physical Society (KNG) in Groningen. At no point during his lecture did he even refer to his own key role in the development of multiscale models.

## Herman the Scientist and Researcher

Whether it was his fascination for sea sailing or not, Herman’s main research interest was and always remained water in biological systems. His PhD thesis from 1962 was an NMR study on the hydration of collagen. In 1967 when I joined his group as an MSc student he was focusing on topics like hydration of DNA by means of NMR, the cloud seeding properties of cholesterol, the influence of water on nerve conduction and the use of microwave dielectric measurements in probing the dynamics of hydrophobic interactions. The latter topic was the subject of the PhD study of Klaas Hallenga and I did my MSc research working together with Klaas on the same subject. Around the time I joined Herman’s group in 1969 the polywater hype based on the findings by Derjaguin and Fedyakin was in full swing. Needless to say that Herman was interested in it. However he very quickly concluded that the “new form of water” was in fact water with silica impurities.

From the early seventies onward he spent every summer several weeks in Orsay, the main location of the Paris-Saclay University. This is where he began his MD studies. I distinctly remember his very enthusiastic presentation of the first modeled open three dimensional box containing 6 × 6 × 6 dynamically interacting and moving water molecules. It was the modest start of what later on was to become the GROMOS model and its successors. Since Herman was spending his time increasingly on modeling biological systems he moved away from research topics based on spectroscopic instruments like NMR and EPR. After George Robillard joined Herman’s group, George took over the responsibility for the latter line of research.

## Herman the Teacher and Educator

Herman had a great interest in teaching but his first courses were not very successful.

He had the bad luck that Professor Jan Kommandeur, his PhD advisor and later senior colleague in the chemistry department, was an outstanding lecturer and teacher. Students would at least subconsciously compare Herman’s teaching to that of Jan. Over time Herman’s teaching skills improved enormously. He was an outstanding mentor in curiosity-driven basic research. Especially when he was personally interested in the topics he was presenting he could be a very motivating advisor. His course on information theory inspired me to write my MSc thesis on the information content of DNA. In the same course he was speculating about the pros and cons of using the entropy concept as a base for taxing pollution and waste associated with industrial production. After entering the field of environmental sciences I have often encountered attempts to use entropy for regulatory purposes in environmental legislation, but invariably these attempts would fail for practical reasons.

Herman was fascinated by the different physiological effects of the noble gases. He gave a convincing imitation of Woody Woodpecker’s sound while telling about the high pitch effects on the human voice after inhaling some helium. But most of all he speculated about the origin of the anesthetic effects of xenon. Through a lucky coincidence during my stay in Boston, I managed to bring together a small team aimed at doing ^129^Xe NMR measurements. This resulted in the first paper on the general anesthetic agent xenon in various chemical and biological environments [3]. The paper would have never seen the daylight without the seeds planted by Herman.

Herman was innovative in his teaching methods. He was experimenting with computer-based teaching long before everybody around him. I remember his small software program aimed at self- teaching the pH concept. His first handouts on error estimation in doing experiments developed over time into “A Student’s guide to Data and Error Analysis” [4].

**Fig. 1:**
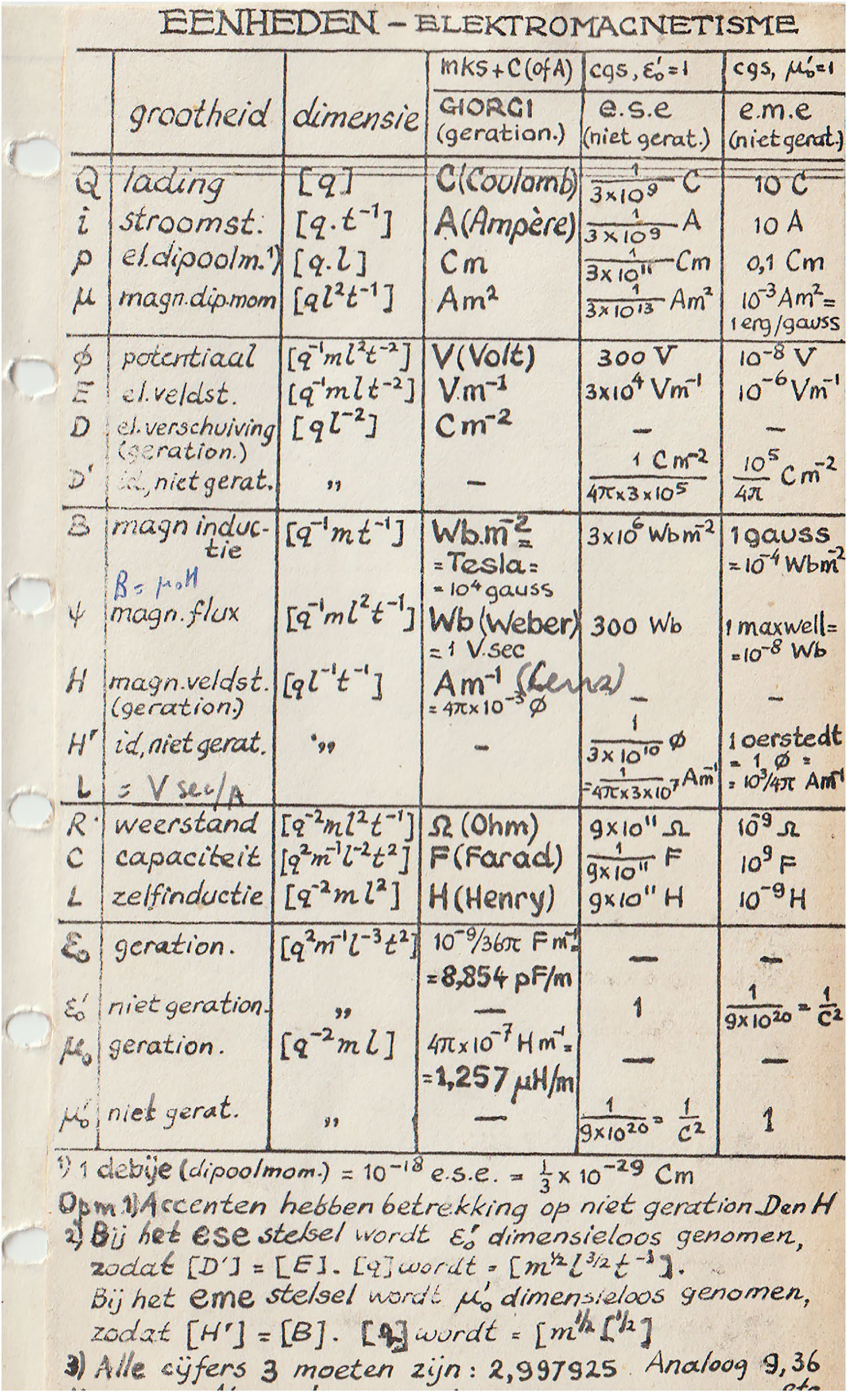
Hand-written handout by Herman Berenden in the format of loose-leaf success agenda

At the end of the sixties he produced a whole series of beautifully hand-written handouts in the format of the pages of the then commonly used loose-leaf Success agenda (diary). The pages contained physical constants, definitions and mathematical expressions and formulas. Luckily I saved some of these pages and Fig. 1 shows one of them. His most impressive written teaching legacy is of course his opus magnum “Simulating the Physical World” [5].

I am very fortunate that Herman was my mentor and advisor at a crucial phase in my career. He truly is and will remain an unforgettable man.
